# Dissolving Microneedles Loaded with Gestodene: Fabrication and Characterization In Vitro and In Vivo

**DOI:** 10.5812/ijpr-131819

**Published:** 2023-05-19

**Authors:** Xueyan Duan, Jianan Ma, Meiying Ning, Yunhua Gao

**Affiliations:** 1 Center of Drug and Medical Polymer Materials, National Research Institute for Family Planning, Beijing, China; 2 Key Laboratory of Photochemical Conversion and Optoelectronic Materials, Technical of Physics and Chemistry, Chinese Academy of Sciences, Beijing, China

**Keywords:** Gestodene, Polyvinylpyrrolidone, Dissolving Microneedle, Sustained Delivery System, Characteristics, Pharmacokinetics

## Abstract

**Background:**

Gestodene (GEST) is widely used in female contraception. It is currently being used as an oral contraceptive. However, unfortunately, oral contraceptives are often associated with several bothersome side effects and poor compliance. Therefore, a sustained delivery system for GEST to overcome these shortcomings is highly desirable.

**Objectives:**

The present study successfully developed a kind of novel dissolving microneedles (DMNs) with a potential for sustained release and a minimally invasive intradermal treatment of GEST.

**Methods:**

The dissolving microneedles containing GEST were fabricated using polyvinylpyrrolidone as the base material. The characteristics in vitro and pharmacokinetics in vivo of GEST-loaded DMNs were investigated.

**Results:**

The results showed that the microneedle could pierce the porcine skin and release the drug at an average dose of 20µg/cm^2^ daily for seven days. The pharmacokinetic experiment of the microneedles indicated that the plasma level of GEST in rats increased with increasing drug dosage, and the plasma drug concentration-time curves were much flatter compared with subcutaneous injection and oral administration. In addition, no cutaneous irritation was observed.

**Conclusions:**

GEST-loaded DMNs may be a promising intradermal sustained delivery system for contraceptive use.

## 1. Background

**Figure 1.**
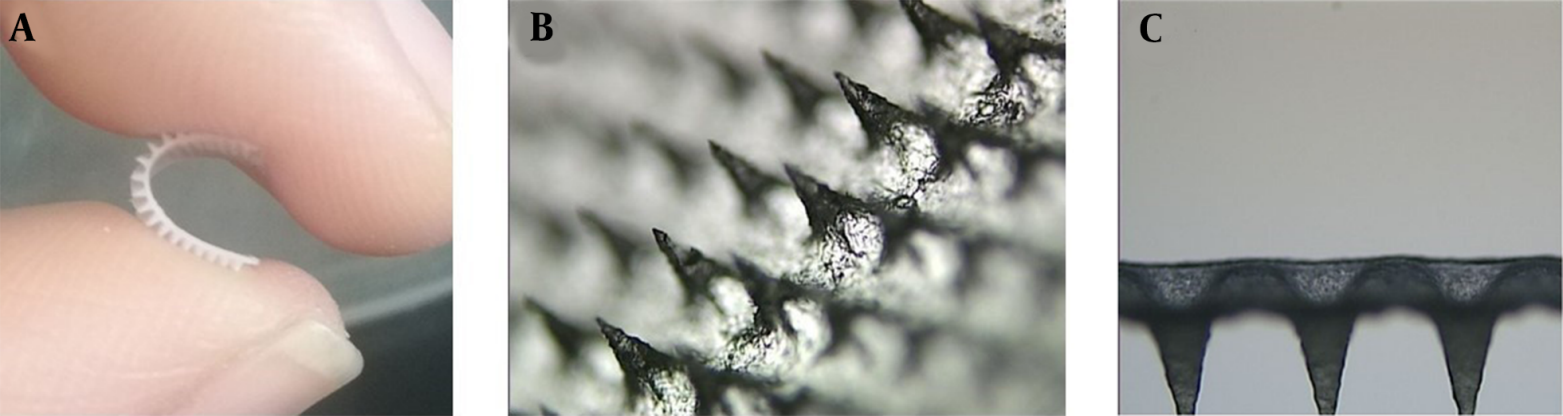
Optical microscope images of GEST-loaded DMNs. (A) flexible images of DMNs, (B) partial images of DMNs, and (C) vertical section images of DMNs.

The solubility of gestodene (GEST) (MW: 310.43, logP_gestodene_: 3.43) (1) in water is 9.11μg/mL (Table 1).The structure of GEST is shown in Figure 1A. GEST is widely used in female contraception as a lone formulation or in combination with estrogen.GEST exhibits good contraceptive properties without any estrogenic and androgenic activities. Currently, the commercially available pharmaceutical preparation of GEST (Minulet^®^) is an oral tablet containing ethinyl estradiol (EE), which is produced by Wyeth. However, bothersome side effects and the inconvenience from daily intake of one tablet of Minulet^®^ results in low compliance (2, 3).

**Table 1.** Solubility of Gestodene in Different Solvents

Solvent	Solubility (μg·mL^-1^)
**Water**	9.11
**PBS (pH7.4)**	7.98
**20% PEG400 (pH7.4)**	43.50
**30% ethanol**	283.52
**50% ethanol**	3253.07

Therefore, non-oral methods of GEST intake have been developed, which include intrauterine contraceptive devices(IUD) (4-6), implants (7), injections (8), intravaginal rings (IVRs) (9), and transdermal patches (10, 11). These offer a long-term effect with good compliance (2, 3). However, the administration of some preparations, including IUD, implants, and injections, require professional skills, which has the disadvantage of inconvenience. In addition, many unexpected adverse events of IVRs, such as leukorrhea, vaginitis, foreign body sensation, and coital problems, can result in higher discontinuation rates. Recently, a novel patch containing GEST and EE has been developed by Bayer Corporation, which can deliver drugs steadily for seven days (12). However, while patches must continuously stay on the skin for seven days, they are subjected to dropping off.

Furthermore, pharmaceutical excipients in patches are irritative to the skin for long-term application, which is unsuitable for people with sensitive skin. Therefore, patches are also inconvenient and find only limited applications. Considering the above-mentioned drawbacks of all materials containing GEST, there is a strong desire to develop convenient and sustained-release contraceptives (13).

Microneedles (MNs) are of minimally invasive drug delivery system that creates micro-scale pores in the skin to increase the permeability of drugs compared to the traditional transdermal patches (14, 15). They are appropriate self-medication (16) and the elders would like to accept MNs administration (17). Over the past years, different kinds of MN arrays have been produced using metals (18-20), glass (21, 22), ceramic (23, 24), silicon (25-29), sugars (30), and polymers (31-33). There are five major types of MNs: Solid (18, 34), drug-coated (29, 34), hollow (20, 21, 35, 36), hydrogel-forming (37, 38), and dissolving microneedles (DMNs) (31, 39-41). Among them, the DMNs fabricated by polymer materials have attracted the most attention from researchers. DMNs are safe because the polymer materials exhibit good biocompatibility and biodegradability (42, 43). Moreover, the drug release of DMNs can be regulated based on the water solubility and the degradability of polymers (44-46). To date, various materials, such as polysaccharides (sodium hyaluronic acid) (47, 48), dextran (49, 50), polymers (polyvinylpyrrolidone, PVP) (31, 51, 52), polyvinyl alcohol (PVA) (53), and Gantrez^®^ copolymers (54), have been used as DMN materials.

## 2. Objectives

In this study, PVP was selected as the base material in the fabrication of DMNs because of its good biocompatibility and dissolution in the skin (55). A kind of new DMNs loading drugs in the MN was prepared using a mold casting technique for the transdermal delivery of GEST. The DMNs loading GEST was convenient and well tolerated for their painless drug delivery system and shorter application time (up to 24h). The procedure of preparation was simple. The characteristics of DMNs were investigated systematically, such as mechanical strength, dissolution rate, and penetration depth. In addition, the pharmacokinetics of GEST-loaded DMNs in rats was examined. The DMNs were a promising alternative drug delivery system for GEST.

## 3. Methods

### 3.1. Materials

GEST (99%) was provided by Zizhu Pharmaceutical Co., Ltd. (Beijing, China). GEST standard (99.4%) and norgestrel (99.4%, internal standard, IS) were purchased from National Institutes for Food and Drug Control (Beijing, China). Polyvinylpyrrolidone (PVP K-90 and PVPK-30) was obtained from Boai NKY Pharmaceutical Ltd (Beijing, China). CMC Na and chondroitin sulfate were obtained from Baichuan biology technology Ltd (Xian, China). Methanol and acetonitrile were of HPLC grade (Fisher Scientific, Geel, Belgium). Trypan blue (TB) was obtained from Sigma-Aldrich (USA). All other chemicals were of analytical reagent grade.

### 3.2. Animals

Female Sprague–Dawley (SD) rats (6 weeks, 200 ± 20g) were purchased from Beijing Wei Tong Li Hua Experimental Animal Technology Co. Ltd (Beijing, China). All animal experiments complied with the Principles of Laboratory Animal Care (NIH) and the demands of the National Act on experiments animals (PR China). The rats were not fed for 12 h but were allowed to drink freely before the experiments.

### 3.3. Fabrication of GEST-loaded DMNs

The MNs were fabricated using a mold casting technique with PVP K90 as the base material. GEST (20.94%, w/w) and PVP (78.53%, w/w) were added to water under magnetic stirring at room temperature. The GEST suspension was filled into the polydimethylsiloxane (PDMS) mold with a vacuum pump. The first layer was dried at room temperature for 2 h. Then, the GEST suspension was filled into the first layer as the DMNs baseplates. All 100 μL of this formulation was filled into the mold according to the abovementioned steps. After drying in a desiccator overnight, the DMNs were detached from the mold. The DMNs containing GEST were examined using optical microscopy. 

### 3.4. In Vitro GEST Release from Drug-loaded DMNs

**Figure 2.**
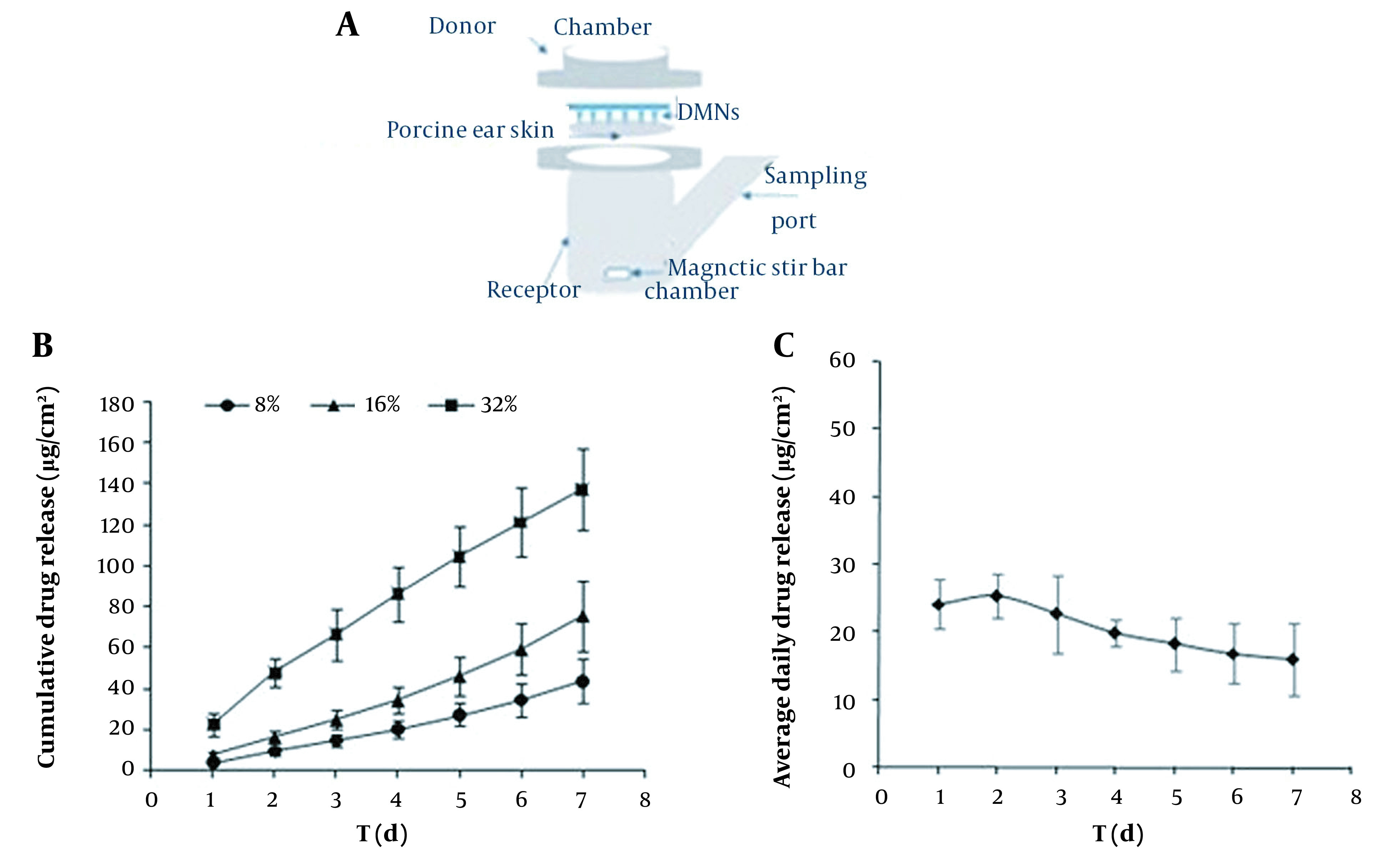
In vitro drug release curves of GEST-loaded DMNs. (A) graphic of a Franz diffusion cell. (B) cumulative drug release from MN patches with different drug loading. (●) 8%, (▲) 16%, (■) 32%. n = 6 ± SD. (C) Daily drug release (32% drug loading). n = 6 ± SD.

The in vitro GEST transdermal release analysis was investigated by Franz diffusion cell (TK-24BL system, Kaikai Technology Co., Ltd., Shanghai, China) with a permeation area of 1.5 cm^2^ (Figure 2A). The subcutaneous fat of the excised porcine (ear) was removed using surgical scissors to obtain a full-thickness skin (0.8 - 1.0 mm thick). The skin was washed with 20% PEG400 PBS (pH = 7.4) before the test to prevent them from drying out. MNs were inserted into the skin samples, which were dried with filter paper. The skin samples with the embedded MNs were then mounted on the Franz cells with a metal horseshoe clip. Based on the results of the determination of the solubility of Gestodene in different media (water, phosphate buffered solution (PBS), 20% PEG400 (pH = 7.4), 30% ethanol, and 50% ethanol), the solubility of the drug in 20% PEG400 (pH = 7.4) satisfies the sink condition of the drug, therefore the in vitro release test selected 20% PEG400 (pH = 7.4) solution as the release medium (Table 1). The receiver compartment was filled with 8mL of 20% PEG400 PBS (pH = 7.4) and retained 37°C in a water incubator. The receptor medium was continuously stirred at 300 rpm. At specified time points (1 d, 2 d, 3 d, 4 d, 5 d, 6 d, 7 d), the samples (n = 6) were taken from the receptor compartments, then an equal volume of fresh and warmed buffer was added. The drug concentrations of samples were analyzed by the high-performance liquid chromatography (HPLC) method to obtain the kinetic parameters and the drug-releasing equation. The mobile phase comprises water (phase A) -acetonitrile (phase B).

### 3.5. Mechanical Test of GEST-loaded DMNs

A force–displacement testing machine (1220SB, Shanghai, China) was used to evaluate the mechanical strength of the GEST-loaded DMNs. The DMNs were put on a small sheet of stainless with needles tip upward. Then the microneedles were pressed by a vertical force of the sensor probe (2×2 mm) at a speed of 1.1 mm/s. The graph of force and displacement was recorded until the needle tips began to fracture. 

### 3.6. Characterization of Skin Penetration In Vitro Pierced by GEST-loaded DMNs

To estimate the skin penetration strength of GEST-loaded DMNs, the DMNs were inserted into the prepared porcine skin. Then the skin was stained with a 5 mg/mL TB solution. After 15 min, the blue spots on the skin were recorded using a digital camera to calculate the penetration ratio. The skin treated with GEST-loaded DMNs was subsequently frozen into an optimum cutting temperature (OCT) compound. The skin samples of frozen sections (10μm thick) were prepared using a cryomicrotome. Some sections were treated with H&E. The holes created by the GEST-loaded DMNs were observed in the images, and their average depth was measured simply by the microscope.

### 3.7. Dissolution Rate of GEST-loaded DMNs in SD Rats

The abdominal hair of pentobarbital-narcotized SD rats was shaved using an electric clipper. The GEST-loaded DMNs were inserted into the skin and removed after 1 and 2 min. Finally, the DMNs before and after insertion were immediately observed using an optical microscope.

### 3.8. In Vivo Study

#### 3.8.1. Experimental Method

The rats were randomly separated into five groups (n = 6): (1) Subcutaneous injection, (2) oral administration, (3) one piece of GEST-loaded DMNs, (4) two pieces of GEST-loaded DMNs, and (5) four pieces of GEST-loaded DMNs. In the s.c. injection and oral administration groups, GEST was suspended in the PVP aqueous solution at a daily dose of 60 µg for six consecutive days. In the DMNs groups, the hair on the abdomen of rats was removed before the experiment. One, two, and four DMNs patches (one patch size: 1 cm^2^, 3.5mg/cm^2^) were respectively applied on the rat skin by a spring applicator. The DMNs patches were removed from the rats’ skin after 24h. Blood samples were taken from the retro-orbital plexus at a predetermined time (0, 2, 4, 8, 12, 24, 48, 72, 96, 120, 144, and 168 h) and centrifuged at 10,000 rpm for 10 min at 4°C.

#### 3.8.2. HPLC-MS/MS Assay for GEST

The GEST concentration in rats was measured using an Agilent 1100 HPLC instrument coupled with a triple quadrupole API 4000 mass spectrometer (Agilent Technologies, USA). The plasma samples above were separated on a Phenomenex™ C18 column (3.0×30 mm) with a particle size of 2.6μm. The samples were eluted by the mobile phase composed of water (phase A) -methanol (phase B) containing 0.1% formic acid under a gradient procedure (Table 2). The flow rate was 800 μL/min. The mass spectrometric conditions were as follows: Ion spray voltage, 5000V; temperature, 550°C; ion source gas1,60psi; ion source gas2, 60psi; curtain gas, 20psi; declustering potential, 106v for GEST and 126v for IS; CE,37v for GEST and 41v for IS; collision cell exit potential, 6v. The positive mode was chosen, and the experiment was performed by multiple reaction monitoring (MRM) using an electrospray ionization source, m/z = 311.125/108.900 and 313.153/109.100 for GEST and IS, respectively.

**Table 2.** HPLC Conditions

Time (h)	A (%)	B (%)
**0.01**	55	45
**0.5**	55	45
**0.8**	5	95
**2.5**	5	95
**2.51**	55	45
**4**	55	45

## 4. Results and Discussion

### 4.1. Characteristics of GEST-loaded DMNs

The degradable polymers such as sodium carboxymethyl cellulose (CMC-Na), chondroitin sulfate, polyvinylpyrrolidoneK30 (PVP K30), and PVP K90 were studied as the DMNs matrix materials. CMC-Na chondroitin sulfate and PVP K30 were unsuitable in preparing DMNs because of their poor moldability and fragility. The DMNs prepared using chondroitin sulfate and PVP K90 had good formability, stiffness, and flexibility. Considering all the above factors, PVP K90 was selected as the optimal matrix due to its good biocompatibility, formability, non-toxicity, and cost-effectiveness.

Upon visual inspection using a stereo microscope, one piece of DMNs comprises 400 (20 × 20) needles on a 1-cm^2^ base plate. Each needle was found to be at a distance of 400.17 ± 15.30 μm from the other and was 500 ± 0.45 μm in height. The MNs are flexible (Figure 1A), which can better fit the skin in practice (54). The MNs were in the shape of quadrangular pyramids (Figure 1B) and sharp and compact (Figure 1C), which showed that the preparation method of DMNs was reasonable.

### 4.2. In vitro Drug Release Kinetics from GEST-loaded DMNs

The in vitro drug release of GEST was evaluated for 7 dayconsecutivlyin Franz diffusion cells, and the results were shown in Figure 2. Figure 2B shows the cumulative drug released into the medium from DMNs with different drug loading (8%, 16%, and 32% w/w). The results indicated that the drug release rate increased upon increasing the drug load. As a result, the DMN patch with 32% drug loading was chosen for further study. The releasing kinetic modeling profiles of DMNs (32% drug loading) in vitro were also compared. Figure 2C showed that the GEST released in vitro from DMNs was sustained for seven days and that the drug amount was approximately 20µg/cm^2^. The most appropriate release model was chosen based on the value of the correlation coefficient of each model (Table 3). This study showed that the formulation was best fitted by the Higuchi model (r = 0.9975) and the zero-order model (r = 0.9969). There are two possible mechanisms: One is the dissolution and degradation of the matrix, and another is the balance between dissolution and diffusion of drug release from the matrix material. Meanwhile, the prolonged release of GEST from DMNs may mainly be due to the high hydrophobicity of the drug, and PVP is just a carrier introducing the drug subcutaneously. The longer half-life of the drug (t_1/2_ = 16h) and the higher concentration of PVP for preparation of microneedles maybe result in its blood drug concentration maintained longer than that of direct subcutaneous injection of drugs.

**Table 3.** Results of Different Equations for the Formulation

	Equation	Correlation (r)
**Zero-order**	y = 17.449x + 13.13	0.9969
**First-order**	y = 0.2017x + 3.4291	0.9334
**Higuchi**	y = 0.0133x + 0.7969	0.9975

### 4.3. Mechanical Properties of GEST-loaded DMNs

**Figure 3.**
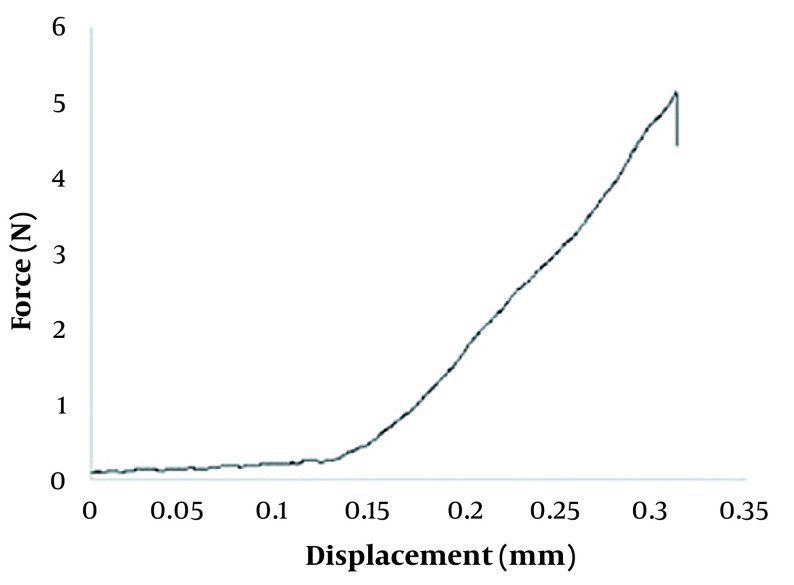
Mechanical behavior of GEST-loaded DMNs.

The force–displacement behavior of GEST-loaded DMNs was recorded using a compression test. As a result, there is no obvious transition point with the increase of the displacement of GEST-loaded DMNs (Figure 3), which indicates that DMNs were not easily broken. The displacement was 0.28 mm under the force of 0.3N/needle, greater than 0.058 N/needle from the previous report (56). Therefore, it may be inferred that the GEST-loaded DMNs have enough hardness and flexibility to penetrate the porcine skin without breakage.

### 4.4. Characterization of Skin Penetration In Vitro Pierced by GEST-loaded DMNs

**Figure 4.**
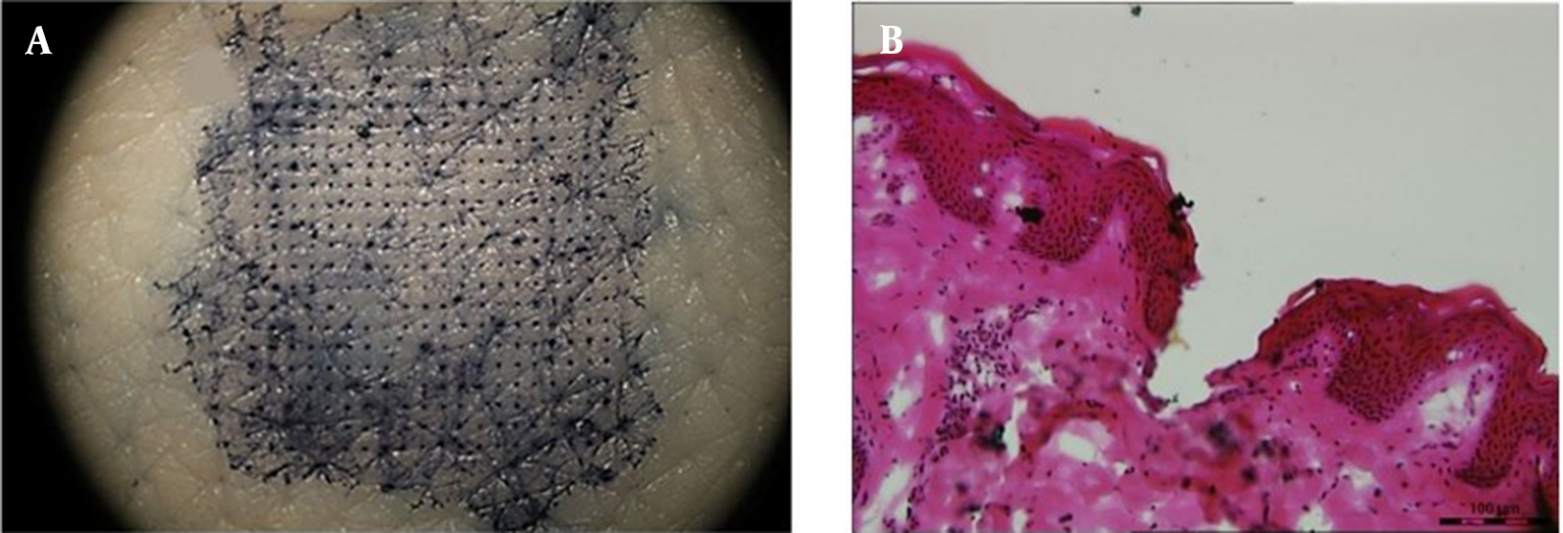
Skin insertion ability of GEST-loaded DMNs. (A) array of drug-loaded DMN holes in porcine skin stained with TB after insertion. (B) histological section of drug-loaded DMNs-treated porcine skin.

As shown in Figure 4A, blue spots are arrayed on the skin. The penetration efficiency of DMNs (the holes stained with TB concerning the whole MNs) was found to be > 90%. The depth of the pores in the porcine skin was simply investigated after applying GEST-loaded DMNs. The channels were shown by histological analysis of the porcine skin sections (Figure 4B), and the insertion depth was calculated as 197 ± 8µm. Therefore, the depth of the pores in the porcine skin was about 200 µm. The elasticity of the skin and the fast dissolution of MNs (42) could be the possible reason for the depth to be far shorter than the height of the MNs (550µm) (32).

### 4.5. Dissolution Rate of GEST-loaded DMNs

**Figure 5.**
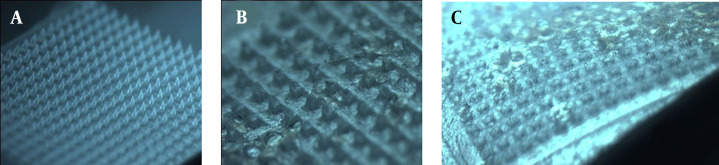
Microscopic images of GEST-loaded DMNs: (A) before, (B) 1 min, and (C) 2 min after application into the skin.

The dissolution process of DMNs before and after piercing into the rat skin was evaluated. As shown in Figure 5, all MNs shafts were completely dissolved within 2 min of application, which is in agreement with the data of PVP as a base solution obtained as reference (30). Rapid dissolution of DMNs could deliver drugs across the skin in a minimally invasive manner, which could greatly improve the compliance of patients.

### 4.6. In Vivo Study

**Figure 6.**
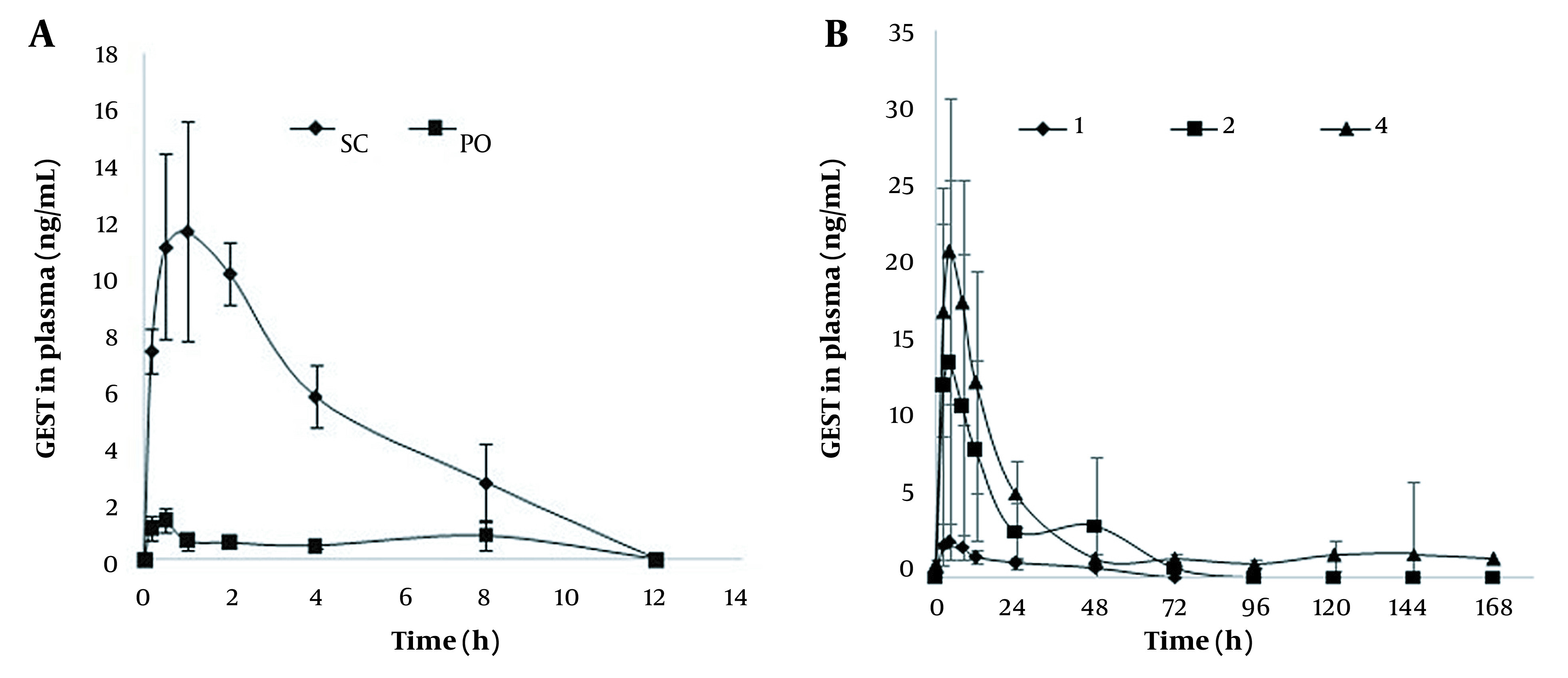
(A) plasma concentration-time profile of GEST after s.c. and p.o. administration of 60 µg GEST daily for 7 days. (B) plasma concentration-time profile of GEST after application of 1 patch, 2 patches, and 4 patches (means ± SD, n = 6).

The plasma profiles of GEST in female SD rats after treatment with s.c. injection, p.o., and DMNs are presented in Figure 6. While GEST could be detected until 48, 72, and 144 h for the DMN groups, GEST could not be detected at 12 h for s.c. injected and p.o. groups. The plasma profiles after the treatment with GEST-loaded DMNs were longer and steadier when compared with the s.c. injection injected and p.o. groups. GEST was deposited in the skin by the drug-loaded DMNs, the drug was sustained and released from GEST-PVP suspension into capillaries, which peaked at 5 h and fell after about 24h, then declined slowly. These results showed that sustained release could be obtained, which agreed with the opinion in the paper (44). As shown in Figure 6B, the peak concentrations of GEST ranged from 3.0 to 21.9 ng/mL after administration of DMNs with three doses, which indicated a significant dose dependency for GEST. 

These results showed that the GEST-loaded DMNs could deliver GEST transdermally for a long time in a minimally invasive way. Importantly, no skin irritation was indicated on any rat after the removal of DMNs.

Table 4 shows the pharmacokinetics of GEST in rats, calculated by DAS 3.2. The T_max_ of administration of DMNs was longer than those of the oral administration and s.c. groups. This might be due to the two procedures of DMNs matrix materials, including absorbance to water and dissolution in the skin, and the solubility of GEST in water was only 9.11 µg/mL. The relative bioavailability of GEST is more than 100%, indicating a higher bioavailability of the drug following the application of GEST-loaded DMNs compared with the oral administration of the pure drug. The relative bioavailability of GEST is also more than 100%, applying 2 and 4 patches of DMNs vs. s.c. administration, which indicating 2 patches of DMNs can be used as the first dose in the clinical trial. Furthermore, the DMNs could increase people’s compliance as DMNs must be worn for 2 min or 1 day at most. Therefore, GEST-loaded DMNs are more convenient than the contraceptive patches (APLEEK, Bayer Corporation), which must be worn for 7 days. All these results show that GEST-loaded DMNs as a new intradermal sustained delivery system is promising for contraceptives.

**Table 4.** Pharmacokinetic Parameters for Plasma GEST Concentration of Rats after the Administration of p.o., s.c. Injection and GEST-loaded DMNs (means ± SD, n = 6)

	AUC (µg/L*h)	T_max_ (h)	C_max_ (µg/L)
**p.o.**	4.9 ± 2.7	0.5 ± 0.3	2.1 ± 1.8
**s.c.**	63.8 ± 60.0	1.2 ± 0.7	12.8 ± 3.4
**1DMN**	33.0 ± 7.4	4.7 ± 2.7	3.0 ± 0.9
**2DMNs**	258.0 ± 187.4	5.0 ± 3.5	14.0 ± 11.6
**4DMNs**	533.7 ± 225.6	4.3 ± 1.9	21.9 ± 9.9

## 5. Conclusions

We successfully developed rapidly dissolving polymeric DMNs containing GEST. The DMNs were fabricated using mold casting technology, and the characteristics in vitro and pharmacokinetics in vivo of the DMNs were evaluated, respectively. The fabrication procedure was very simple. With good mechanical strength and flexibility, the GEST-loaded DMNs could effectively pierce the stratum corneum to deliver GEST into the skin. The dissolution data in vitro was fitted to the Higuchi equation (r = 0.9975) and zero-order equation (r = 0.9969), which could reduce the fluctuation of drug concentration. Compared with s.c. injection and oral administration, a longer plasma level of GEST was achieved. In addition, the needles dissolved fast, and there was no irritation to the rat skin. In conclusion, the method offers a potential and convenient route for long-acting contraceptives compared to oral pills. To ensure the safety and reliability of GEST-loaded DMNs in clinical use, further studies are needed.
